# Predicting amyloid positivity in patients with mild cognitive impairment using a radiomics approach

**DOI:** 10.1038/s41598-021-86114-4

**Published:** 2021-03-26

**Authors:** Jun Pyo Kim, Jonghoon Kim, Hyemin Jang, Jaeho Kim, Sung Hoon Kang, Ji Sun Kim, Jongmin Lee, Duk L. Na, Hee Jin Kim, Sang Won Seo, Hyunjin Park

**Affiliations:** 1 Department of Neurology, Samsung Medical Center, School of Medicine, Sungkyunkwan University, Seoul, South Korea; 2 Samsung Alzheimer Research Center, Samsung Medical Center, Seoul, Korea; 3 Neuroscience Center, Samsung Medical Center, Seoul, Korea; 4 Department of Electronic and Computer Engineering, Sungkyunkwan University, Suwon, Korea; 5 Department of Neurology, Dongtan Sacred Heart Hospital, Hallym University College of Medicine, Hwaseong, Korea; 6 Center for Neuroscience Imaging Research, Institute for Basic Science, Suwon, Korea; 7 School of Electronic and Electrical Engineering, Sungkyunkwan University, Suwon-si, Republic of Korea; 8 Department of Health Sciences and Technology, SAIHST, Sungkyunkwan University, Seoul, Korea; 9 Department of Clinical Research Design and Evaluation, SAIHST, Sungkyunkwan University, Seoul, Korea; 10 Center for Clinical Epidemiology, Samsung Medical Center, Seoul, Korea; 11 Department of Intelligent Precision Healthcare Convergence, Sungkyunkwan University, Suwon-si, Korea

**Keywords:** Computational biology and bioinformatics, Neuroscience, Neurology, Biomarkers

## Abstract

Predicting amyloid positivity in patients with mild cognitive impairment (MCI) is crucial. In the present study, we predicted amyloid positivity with structural MRI using a radiomics approach. From MR images (including T1, T2 FLAIR, and DTI sequences) of 440 MCI patients, we extracted radiomics features composed of histogram and texture features. These features were used alone or in combination with baseline non-imaging predictors such as age, sex, and ApoE genotype to predict amyloid positivity. We used a regularized regression method for feature selection and prediction. The performance of the baseline non-imaging model was at a fair level (AUC = 0.71). Among single MR-sequence models, T1 and T2 FLAIR radiomics models also showed fair performances (AUC for test = 0.71–0.74, AUC for validation = 0.68–0.70) in predicting amyloid positivity. When T1 and T2 FLAIR radiomics features were combined, the AUC for test was 0.75 and AUC for validation was 0.72 (*p* vs. baseline model < 0.001). The model performed best when baseline features were combined with a T1 and T2 FLAIR radiomics model (AUC for test = 0.79, AUC for validation = 0.76), which was significantly better than those of the baseline model (*p* < 0.001) and the T1 + T2 FLAIR radiomics model (*p* < 0.001). In conclusion, radiomics features showed predictive value for amyloid positivity. It can be used in combination with other predictive features and possibly improve the prediction performance.

## Introduction

Since the concept of a biomarker-based biological definition of Alzheimer’s disease (AD) is now widely accepted among researchers and clinicians, testing the presence of amyloid pathology in the brain is becoming more important. Amyloid β peptide (Aβ) status is crucial not only for diagnostic purposes, but also for predicting the clinical course of patients in the early stage of AD. In particular, Aβ status is associated with clinical deterioration and transition to dementia in patients with mild cognitive impairment (MCI)^1,2^. There are two commonly used biomarkers for Aβ pathology: cerebrospinal fluid Aβ42 level and amyloid positron emission tomography (PET). Amyloid PET is a non-invasive technology that is becoming widely available and may have higher reliability in longitudinal examinations and between centers than CSF measures^3^. However, like all PET techniques, its clinical application has been limited due to non-medical factors, such as high cost, limited availability, and patients’ concerns for radiation exposure.

Most recent clinical trials regarding the development of disease-modifying treatment for AD target amyloid-positive mild cognitive impairment (MCI) patients. There is some consensus among researchers that when a patient becomes demented, it might be too late for any intervention. However, targeting MCI makes it difficult to select amyloid positive subjects. Screening failure rates in clinical trials for MCI due to AD are very high (up to 80%)^4^. Among the causes of screening failure, one of the most frequent was Aβ negativity on amyloid PET. This is primarily because only about 40–60% of patients with MCI are amyloid positive^5^. High screening failure rates also hinder the efficient use of resources in clinical trials. Therefore, both clinically and in research, discriminating MCI subjects with a high probability of having cerebral amyloid deposits may be beneficial.

There have been efforts made to predict amyloid positivity of MCI subjects using more widely available modalities such as neuropsychological tests, ApoE genotype and brain magnetic resonance imaging (MRI). In our previous study, we developed a nomogram for predicting amyloid positivity using neuropsychological test results and ApoE genotype^6^. The performance of the model was at the fair level (AUC = 0.77). Many studies trying to predict amyloid positivity have shown similar accuracy. Among the studies where a cross-validation or external validation scheme was applied, classification performances were between fair to good levels^7–11^.

To incorporate brain MRI findings into a prediction model, features which characterize the image should be determined. In AD research, morphometric features such as cortical thickness, hippocampal or grey matter volume and structural deformity have been commonly used. Texture features and the characterization of changes in local patterns of signal intensity, have been also used in studies regarding AD^12–15^. Radiomics is a high-dimensional quantitative feature analysis approach, which can compute a set of features that uniquely characterize the target region of interest (ROI)^16–18^. The high-dimensional features in the radiomics approach include histogram-based features as well as texture features. While radiomics has been widely used in oncology research, only recently has it been applied to AD research^19^. A recent study explored radiomics features and learned features from a deep neural network to classify AD from healthy controls using MRI^20^. Some studies revealed that a high-level latent feature learned from deep neural network could be potential tool for the diagnosis of AD and its prodromal stage, MCI^21–23^. Most studies using texture analysis or radiomics approaches have focused on the discrimination of diagnostic groups or the prediction of conversion to dementia^24–26^.

In the present study, we developed a prediction model of amyloid positivity in MCI patients using a well-established approach of radiomics. While previous studies utilized macroscale changes in brain MRI, levels^7,8,27^. we aimed to test whether these mesoscale features can classify amyloid status. We compared the predictive values of radiomics-based prediction models using different MRI sequences. We also compared the predictive value of a radiomics model with those of cortical thickness and non-imaging predictors such as demographic information (age, sex) and ApoE genotype and tested whether the combination of those modalities would benefit the prediction model. We hypothesized that we would be able to improve predictive performance by combining information from radiomics and non-imaging features.

## Results

### Clinical characteristics

Scans of 440 subjects (348 subjects in the training/test set, 92 subjects in the validation set) were included in the final analysis. The characteristics of the subjects are shown in Table 1, and Supplementary Table 1. The mean (SD) age of all participants was 71.3 (8.3) years range. One hundred fifty-three subjects (44.0%) were APOE e4 carriers, and 166 subjects were positive for amyloid as assessed with PET. There was no significant difference of age, sex, educational attainment, and mean cortical thickness between amyloid positive and negative subjects. However, Amyloid-positive subjects had poor cognition [MMSE score, 26.5 (Aβ (−)) vs. 25.4 (Aβ (+)), *p* = 0.001; CDR-SOB, 1.38 (Aβ (−)) vs. 1.89 (Aβ (+)), *p* < 0.001] compared to amyloid-negative subjects. The mean (SD) global SUVR of ^18^F-Florbetaben (reference: whole cerebellum) was 1.01 (0.14) in the amyloid negative group and 1.53 (0.21) in the positive group. The mean (SD) ^18^F-Flutemetamol global SUVR (reference: whole cerebellum) was 1.00 (0.18) and 1.60 (0.20) in amyloid negative and positive group, respectively. Direct conversion of FBB and FMM SUVRs into centiloid units^28^ showed that the mean (SD) centiloid units of ^18^F-Florbetaben was 11.9 (24.6) in the amyloid negative group and 85.6 (35.9) in the positive group. The mean (SD) centiloid unit of ^18^F-Flutemetamol SUVR was 27.2 (43.3) and 84.0 (44.5) in amyloid negative and positive group, respectively.

**Table 1:** Clinical characteristics of participants (N = 348).

	Overall (N = 348)	Amyloid negative (N = 182)	Amyloid positive (N = 166)	*p* value
Age, mean (SD), years	71.3 (8.3)	70.9 (8.6)	71.9 (8.1)	0.279
Male sex, No. (%)	148 (42.5)	78 (42.9)	70 (42.2)	0.983
Education, mean (SD), years	12.0 (4.7)	12.0 (4.8)	11.9 (4.6)	0.931
APOE e4 carrier, No. (%)	153 (44.0)	44 (24.2)	109 (65.7)	< 0.001
MMSE score, mean (SD)	26.0 (3.0)	26.5 (2.8)	25.4 (3.1)	0.001*
CDR-SOB, mean (SD)	1.62 (1.08)	1.38 (1.00)	1.89 (1.13)	< 0.001*
Mean cortical thickness, mean (SD), mm	2.42 (0.11)	2.42 (0.10)	2.42 (0.11)	0.927^†^

Data are presented as mean (standard deviation) for continuous variables and N (%) for categorical variables.

*MMSE* mini-mental state examination, *CDR-SOB* Clinical Dementia Rating Scale Sum of Boxes.

*p values were obtained from linear models, corrected for age, sex, and educational attainment.

^†^*p* values were obtained from linear models, corrected for age, sex, and intracranial volume.

### Performance in amyloid PET positivity prediction

**Figure 1:**
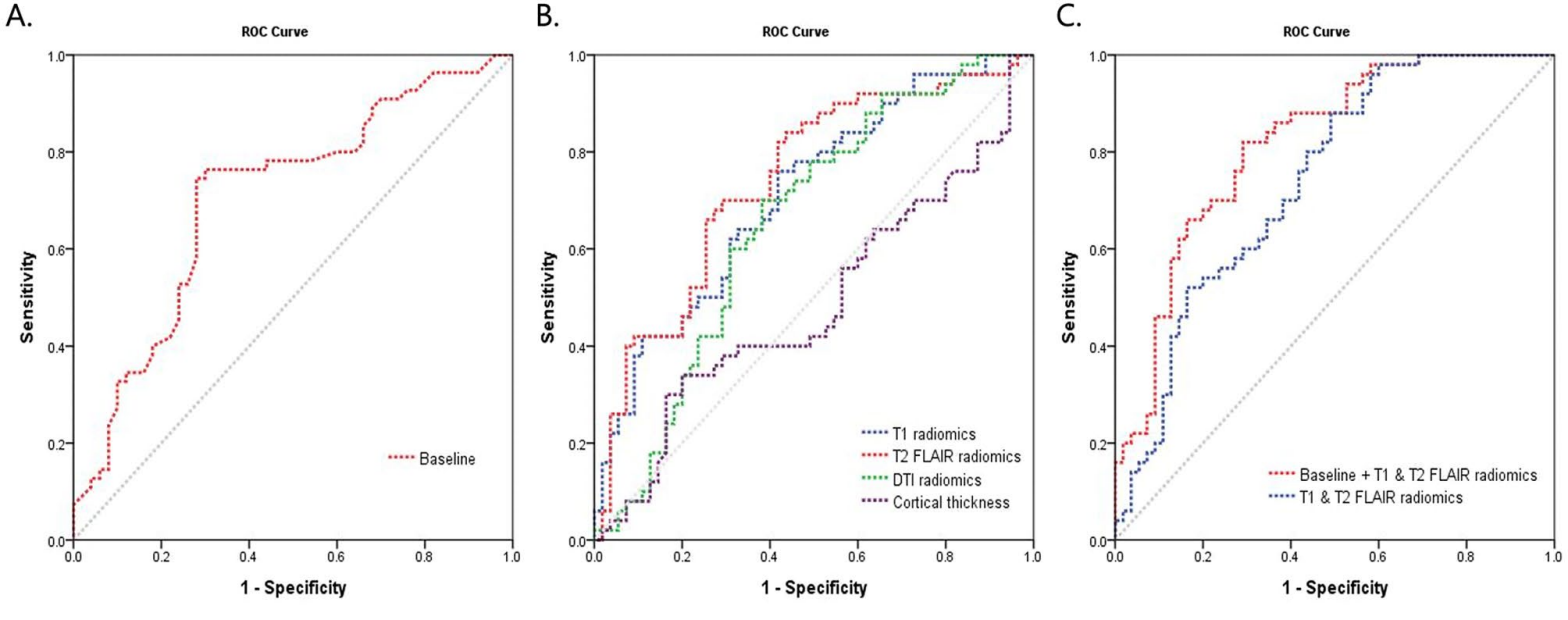
Receiver operative characteristics curves of prediction models. Curves from prediction models using baseline features (**A**), features from single MRI sequence (**B**), and combined features (**C**) were plotted.

The estimated performance of the classifiers with baseline predictors (age, sex, and ApoE genotype) alone, radiomics features from each MRI modality, as well all combinations are shown in Table 2. The prediction models were built using a bootstrap approach where the original training set was split into 70% training and 30% testing 100 times maintain the ratio of amyloid positive and negative cases. In each iteration, the model building procedure repeated and the constructed model was subsequently applied to testing and validation sets for performance evaluation. The performance of the baseline model was fair (AUC = 0.71, sensitivity = 0.74, specificity = 0.69). Among models using features from single MRI modality, the model using cortical thickness did not perform better than chance (AUC 0.49, 95% confidence interval 0.48–0.49). Therefore, cortical thickness features were not included in further analysis. Within other single-modality models, the T2 FLAIR radiomics model showed the highest performance in the test sets (AUC for test = 0.74) in predicting amyloid positivity, although it performed worse than baseline model in the validation set (AUC for validation = 0.68). When T1 and T2 FLAIR radiomics features were combined, the AUC for test was 0.75 (sensitivity = 0.75, specificity = 0.69) and the AUC for validation was 0.72 (sensitivity = 0.58, specificity = 0.80), which were significantly higher than that of the baseline model (*p* < 0.001). Also, when compared to the models using single sequence radiomics features, the AUC was higher compared to all models (*p* = 0.036 for T2 FLAIR model, *p* < 0.001 for T1, DTI models). When baseline features were combined with the T1 and T2 FLAIR radiomics model, the model showed the best performance (AUC for test = 0.79, sensitivity = 0.65, specificity = 0.83; AUC for validation 0.76, sensitivity 0.68, specificity 0.75) amongst evaluated models, which was significantly better than the baseline model (*p* < 0.001) and the T1 + T2 FLAIR radiomics model (*p* < 0.001). The ROC curves for prediction models are shown in Fig. 1. Additional results of considering wavelet features are given in the Supplement.

**Table 2:** Performances of the prediction models.

	AUC	Sensitivity	Specificity	*p* (vs. T1 and FLAIR)
A. Test sets				
Baseline model	0.71 (0.71–0.72)	0.74 (0.73–0.75)	0.69 (0.67–0.70)	< 0.001
Single MRI modality				
T1 radiomics	0.71 (0.70–0.72)	0.73 (0.70–0.76)	0.63 (0.61–0.66)	< 0.001
T2 FLAIR radiomics	0.74 (0.73–0.75)*	0.69 (0.67–0.71)	0.73 (0.71–0.75)	0.036
DTI radiomics	0.65 (0.65–0.66)	0.76 (0.74–0.79)	0.57 (0.55–0.59)	< 0.001
Cortical thickness	0.49 (0.48–0.49)	0.07 (0.03–0.10)	0.95 (0.93–0.98)	< 0.001
Combined models				
T1 and T2 FLAIR radiomics	0.75 (0.75–0.76)*	0.75 (0.73–0.77)	0.69 (0.67–0.71)	–
Baseline + T1 and T2 FLAIR	0.79 (0.79–0.80)*	0.65 (0.63–0.67)	0.83 (0.82–0.85)	< 0.001
B. Validation set				
Baseline model	0.70 (0.70–0.70)	0.79 (0.79–0.79)	0.57 (0.57–0.57)	< 0.001
Single MRI modality				
T1 radiomics	0.70 (0.69–0.70)	0.52 (0.50–0.54)	0.82 (0.80–0.84)	< 0.001
T2 FLAIR radiomics	0.68 (0.68–0.69)	0.74 (0.71–0.77)	0.61 (0.58–0.64)	< 0.001
DTI radiomics	0.62 (0.62–0.63)	0.56 (0.54–0.59)	0.68 (0.66–0.70)	< 0.001
Cortical thickness	0.49 (0.48–0.49)	0.09 (0.05–0.13)	0.94 (0.90–0.97)	< 0.001
Combined models				
T1 and T2 FLAIR radiomics	0.72 (0.71–0.72)*	0.58 (0.55–0.61)	0.80 (0.77–0.81)	–
Baseline + T1 and T2 FLAIR	0.76 (0.76–0.77)*	0.68 (0.64–0.71)	0.75 (0.73–0.78)	< 0.001

95% confidence intervals are presented in brackets.

*AUC* area under the curve, *ApoE* apolipoprotein E genotype, *MRI* magnetic resonance imaging, *FLAIR* fluid attenuation inversion recovery, *DTI* diffusion tensor imaging.

*Significantly higher than baseline model (*p* < 0.001).

### Frequently selected features

Frequently selected features, defined by being selected more than 50 times during the 100 bootstrapping, are shown in Supplementary Table 4. Among the baseline features, only the ApoE genotype was consistently selected when combined with the T1 and T2 FLAIR radiomics features in the model, while age and sex were not. In terms of imaging features, the features from the T2 FLAIR images tended to be selected most consistently, while DTI features were rarely selected. In single MRI sequence models without baseline features, while 21 features were selected consistently in the T2 FLAIR model, only two features were consistently selected in the DTI model.

## Discussion

In this study, we evaluated several models for predicting amyloid positivity in MCI patients. The models were built using combinations of non-imaging features and radiomics features from MRI modalities. T2 FLAIR- and T1 Radiomics-based models showed potential as imaging biomarkers for predicting the amyloid positivity with fair accuracy. When radiomics features from both sequences were combined with basic clinical information, the prediction performance was improved significantly. Thus, our findings suggest that the combined model, which uses radiomics features as well as routine clinical variables (i.e., ApoE genotype), could provide a better prediction of disease status for MCI patients. Furthermore, our results might save resources in clinical trials by reducing the screening failure rate. This model can reduce the number needed to screen from 2.10 to 1.29, resulting in a reduction of the cost for amyloid PET by 1.4 million US Dollars when recruiting 500 amyloid positive MCI patients assuming a conservative estimate of 3500 Dollars per scan.

Our first major finding was that even when radiomics features from a single sequence were used, the prediction model showed fair performance. While the model using cortical thickness showed poor performance which was similar to chance, those using radiomics features from T2 FLAIR (AUC = 0.74) or T1 weighted images (AUC = 0.71) showed fair performances. This result is consistent with the findings of previous reports which found microstructural changes precede macroscopic atrophy^29^. Results from previous histological studies suggest that T1 weighted images can reflect neuronal density^30,31^, while T2-weighted images reflect myelination^31^. Although interpretations of what the radiomics features reflect should be made with caution without pathological data, amyloid-beta associated with neuronal degeneration may have resulted in subtle alteration in T1 or T2 FLAIR signal intensity.

Our second major finding was that using radiomics features from both T1 and T2 FLAIR sequences for amyloid prediction increased the prediction performance. It is known that the ratio between the signal intensity of the T1- and T2-weighted images is known to reflect cortical myelin content^32^. A recent study has shown that cortical T1/T2 signal intensity ratio significantly differed between AD patients and normal controls^33^. Although we did not include the T1-weighted and T2 FLAIR signal intensity ratio, including features from both sequences might have increased the ability of the prediction model to reflect the microstructural alterations more accurately.

We also found that incorporating non-imaging features to the prediction model improved its performance. In fact, the model using non-imaging features, including age, sex, and ApoE genotype showed similar performance compared to the models using imaging features. This result is in line with previous reports, showing fair to good performances of non-imaging variables, particularly ApoE genotype. The AUC values reported in amyloid positivity prediction with ApoE genotype ranged from 0.72 to 0.81^10,27^. In the present study, the model encompassing both non-imaging features and the T1 and T2 FLAIR radiomics features resulted in the highest performance among the models tested. It predicted amyloid positivity significantly better than both the baseline model and the T1 and T2 FLAIR radiomics model.

It is noticeable that among the three non-imaging features (age, sex, and ApoE genotype), only the ApoE genotype was selected consistently in the LASSO feature selection. By contrast, despite the known fact that the occurrence of brain amyloidosis rises with aging in healthy people^34^, age did not have a significant role in our models. Unlike in healthy controls, the prevalence of amyloid positivity does not vary according to age in clinically diagnosed AD patients^34^. This difference among groups may have resulted in the non-significant effect of age in our models. Regarding the effect of sex on AD pathology, a previous neuropathological study showed that while women had more neurofibrillary tangles, there was no difference in the amount of amyloid pathology between men and women^35^. A meta-analysis also showed that there was no difference in amyloid positivity between sexes^36^. Our finding that sex didn’t have a significant role in amyloid prediction is in line with these previous studies.

There have been efforts to predict amyloid positivity in MCI patients using MRI images. Of those studies, one group used anatomical shape variations using T1-weighted images for predicting amyloid positivity and achieved the highest prediction performance (AUC = 0.88)^27^. Performances of other studies ranged between fair to good levels^7–11^. Diffusion tensor imaging has also been used to predict prodromal AD among MCI patients, although it did not show good performance (accuracy = 0.68)^7^. Although various attempts have been made using different predictive variables, there have been no reports predicting amyloid positivity using radiomics features. In fact, radiomics approaches have mainly been utilized in the oncology field, and it is just being applied to the field of neurodegenerative diseases^37^. Although our prediction models did not outperform previously reported models, the results imply the potential value of the radiomics approach in AD research.

Our findings imply that combining radiomics models from imaging with conventional clinical variables was effective at predicting amyloid positivity. In general, clinical variables are associated with whole-brain level changes, while radiomics features can detect minute changes at the pixel level perhaps modeling mesoscale changes. Thus, radiomics feature could be providing more fine-grained local information of AD-related tissue-level changes. Therefore, combining two scales of information, one from clinical variables (macroscale) and the other from radiomics features (mesoscale), could be complementary and hence might lead to improved model performance in our study.

Taken together, we developed prediction models for amyloid positivity in MCI patients using a radiomics approach, with a larger sample size compared to those in previous reports. However, this study has some limitations. First, although we adopted a bootstrapping approach for performance estimation, the results were not externally validated in an independent cohort. Further studies using larger samples possibly in a multi-center setting are needed. Second, our features do not provide topographic information in terms of contribution to the classification. This, however, is an inherent characteristic of texture features as opposed to morphometric features. Third, while pathological changes of AD occur throughout whole brain, radiomics features used in this study only capture the changes within the pre-defined ROIs. Although precuneus and hippocampus are crucial structures showing early changes in AD, other structures involved in AD pathological process need to be studied in the future study. Fourth, participants with MCI in our dataset underwent two different ^18^F-labelled amyloid PET or different methods in defining Aβ positivity. However, this limitation might be mitigated by previous results showing that the accuracies of visual assessment and quantitative assessment in evaluating Aβ positivity were comparable^38,39^. In addition, our recent study investigated the concordance rate for Aβ positivity between florbetaben (FBB) and flutemetamol (FMM) in 107 participants who underwent both FBB and FMM PET for Aβ deposits. High agreement rates were found between FBB and FMM in visual assessment (94.4%) and SUVR cut-off categorization (98.1%). In addition, both FBB and FMM showed high agreement rates between visual assessment and SUVR cut-off categorization (93.5% in FBB and 91.6% in FMM)^40^. Fifth, selecting grey matter regions as ROI might have made the comparison between DTI features and others unfair. Although less studied, DTI has been used not only to analyze nerve tracts in white matter but also to measure microstructural alterations in gray matter. Some study results showed that DTI findings in hippocampus predicted conversion to dementia^41^ and also had diagnostic utility in AD^42^. Furthermore, there are some evidences that mean diffusivity is also altered in cerebral cortex^43,44^. Sixth, since we used the global cortex to extract cortical thickness features and only two predefined ROIs for radiomics features, the comparison between cortical thickness and radiomics features might not reflect each modality’s full potentials. Seventh, deep learning could improve the performance of the prediction. However, it comes at a cost of high sample size requirements. We hope to explore deep learning approaches in future studies as we collect more data. Finally, since we do not have pathological data, the interpretation of radiomics features is limited. Nevertheless, it is noteworthy that we adopted the radiomics approach in the prediction model for amyloid positivity and achieved acceptable performance.

In conclusion, radiomics features composed of histogram features and texture features have predictive value for amyloid positivity. The performances of the models were at least comparable to previously reported models using imaging features. This radiomics approach can be used in combination with other predictive features and can possibly improve the prediction of amyloid positivity in patients with MCI.

## Methods

### Participants

A total of 446 subjects with amnestic MCI (aMCI) who underwent amyloid PET scans between August 2015 and March 2018 were recruited from the in-house PET registry of the Samsung Medical Center (Seoul, Korea), which were used as training and testing sets in a bootstrap fashion. Additional 103 subjects were recruited between August 2019 and June 2020, which were used as an independent validation set. Patients who underwent a full neuropsychiatric battery, APOE genotyping, and multimodal MRI, including 3-Dimensional T1-weighted, T2 FLAIR, and diffusion tensor imaging (DTI) were included. Twenty-two subjects missing 3-Dimensional T1 images, and 69 subjects missing DTI, and five subjects with image processing error were excluded. Thirteen subjects without APOE genotype information were also excluded. As a result, a total of 440 subjects (348 subjects in the training and test sets, 92 subjects in the validation set) were included in our analysis. All patients were in accordance with Petersen’s clinical criteria for MCI^45^ with the following modifications: (1) subjective memory problems reported by the patient or caregiver, (2) normal activities of daily living (ADL) as judged by an interview with a clinician and Seoul–Instrumental ADL test (with a score < 8)^46^, (3) objective memory decline below the 16th percentile or the norm determined by neuropsychological tests, and (4) no dementia. All subjects were evaluated with comprehensive interviews, neurological examinations, and neuropsychological assessments. Blood tests to exclude secondary causes of dementia included a complete blood count, blood chemistry tests, vitamin B12/folate levels, syphilis serological tests, and thyroid function tests. Conventional brain MRI scans confirmed the absence of structural lesions such as tumors, traumatic brain injuries, hydrocephalus, or severe white matter hyperintensities.

This study was approved by the Institutional Review Board of Samsung Medical Center, and informed consent was obtained from the patients and caregivers. All research was conducted in accordance with the principles of the Declaration of Helsinki.

### PET acquisition and interpretation

Subjects underwent ^18^F-florbetaben PET or ^18^F-flutemetamol PET to detect amyloid in the brain. According to protocols proposed by the ligand manufacturers, a 20 min emission PET scan with dynamic mode (consisting of 4 × 5 min frames) was performed 90 min after injection of a mean dose of 311.5 MBq FBB and 185 MBq FMM, respectively. PET images were rated as amyloid positive or negative by a nuclear medicine physician. ^18^F-florbetaben PET was defined as positive when visual assessment was scored as 2 or 3 on the brain Aß plaque load (BAPL) scoring system^47^. Visual interpretation of ^18^F-flutemetamol PET images relied upon a systematic review of five brain regions (frontal, parietal, posterior cingulate and precuneus, striatum, and lateral temporal areas). If any of the brain regions were positive in either hemisphere, the scan was considered positive^48^.

### MRI acquisition

We acquired 3-dimensional T1 turbo field echo (TFE), 3-dimensional fluid-attenuated inversion recovery (FLAIR), and diffusion tensor imaging sequences using 3.0T MRI scanners (Philips, Best, the Netherlands). The MR imaging was performed following the protocol described in our previous work^49,50^. The 3-dimensional T1 MRI data were acquired using the following imaging parameters: sagittal slice thickness, 1.0 mm with 50% overlap; no gap; repetition time of 9.9 ms; echo time of 4.6 ms; flip angle of 8°; and matrix size of 240 × 240 pixels reconstructed to 480 × 480 over a field of view of 240 mm. In whole-brain DTI-MRI examination, sets of axial diffusion-weighted single-shot echo-planar images were collected with the following parameters: 128 3 128 acquisition matrix; 1.72 3 1.72 3 2 mm^3^ voxel size; 70 axial slices; 22 3 22 cm^2^ field of view; echo time 60 ms, repetition time 7,696 ms; flip angle 90°; slice gap 0 mm; b-factor of 600 s/mm^2^. Diffusion-weighted images were acquired from 45 different directions using the baseline image without weighting (0, 0, 0). All axial sections were acquired parallel to the anterior commissure–posterior commissure line.

### Image preprocessing

**Figure 2:**
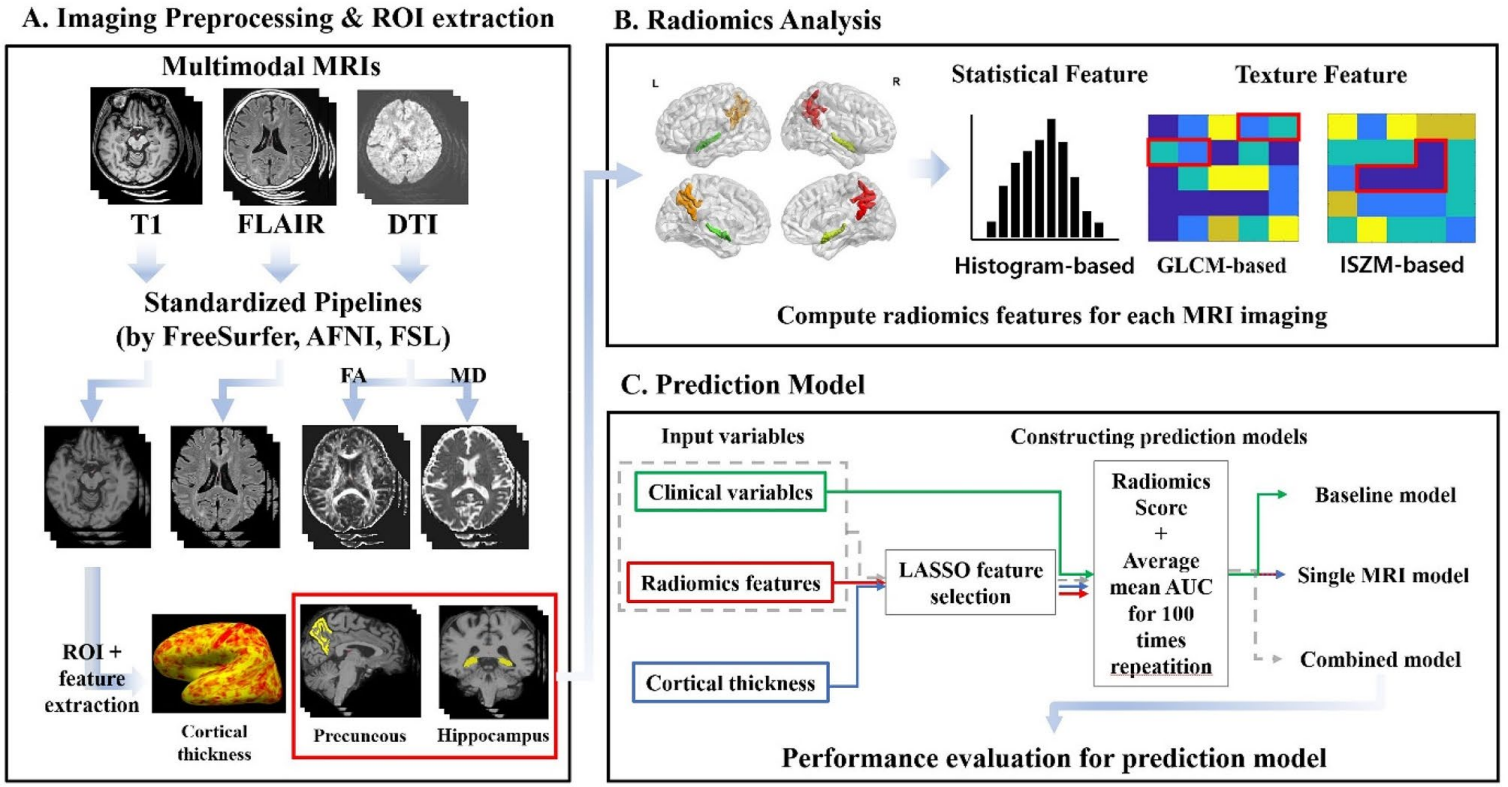
Overview of image processing and modeling. Schematic figures of image preprocessing and ROI extraction pipeline (**A**), radiomics analysis within precuneus and hippocampal ROIs (**B**), prediction model development using clinical and imaging features (**C**).

T1-weighted, FLAIR and DTI data were preprocessed using standardized pipelines (Fig. 2A). T1-weighted MRI was processed using the recon-all function in FreeSurfer^51–56^ as follows: In volumetric space, the magnetic field inhomogeneity correction, non-brain tissue removal, intensity normalization, and whole brain parcellation were performed. Next, white (located between white and gray matter) and pial (located between gray matter and cerebrospinal fluid) surfaces were generated and averaged to construct a mid-thickness surface, which was used to generate a spherical surface. The spherical surface was registered onto a 164k vertex mesh and then down-sampled to a 32k vertex mesh. In this study, we used the parcellated cortical areas provided by FreeSurfer as region of interests (ROIs). Cortical thickness obtained from FreeSurfer was also used as an imaging feature. FLAIR data was processed using AFNI software^57^ as follows: The orientation of data was matched with standard data, magnetic field inhomogeneity was corrected for, and non-brain tissues were removed. DTI data was processed using FSL software^58^. The skull stripped data was corrected for distortions induced by eddy current and head motion. DTI data were then reconstructed based on the corresponding gradient table using DTIFIT function in FSL to calculate functional anisotropy (FA) and mean diffusivity (MD).

### Radiomics analysis and building prediction models

For radiomics analysis, we focused on two ROIs, the hippocampus and precuneus, from preprocessed results by FreeSurfer. Precuneus is known as one of the regions that show amyloid accumulation in the earliest phase of Alzheimer’s disease^59^. Also, in terms of structural atrophy, the hippocampus is one of the earliest affected sites^60,61^. These ROIs were registered onto the native space for three MRI modalities, respectively. Radiomics features were then computed for three modalities (Fig. 2B). We computed 144 radiomics features for two ROIs (left-hippocampus, right hippocampus, left precuneus, and right precuneus) using PyRadiomics software^62^. Full details of the features are given in the Supplement. For DTI data, we considered 288 radiomics features as we computed features from FA and MD images. Three categories of radiomics features were considered: histogram-based features, gray-level co-occurrence matrix (GLCM)-based, and intensity size zone matrix (ISZM)-based features. Histogram-based features are first-order statistics from the distribution of intensity values within ROI. GLCM-based and ISZM-based features are texture-based features, which reflect the spatial or regional relationships between adjacent voxels. Specifically, GLCM-based features represent how often a voxel with a specific intensity value occurred in a specific spatial relationship with a neighboring voxel^63^. ISZM-based features considered how voxels with a similar intensity value occurred with varying cluster size^64,65^. As a part of supplementary analysis, we also computed wavelet-based features by calculating radiomics features on high/low pass filtered images in eight combinations.

Computed radiomics features were used to construct models which aimed to predict amyloid positivity in patients. We performed feature selection using the least absolute shrinkage and selection operator (LASSO) regression method to select a few features which predicted outcome for amyloid pathology assessment (i.e., amyloid positivity). This procedure enhances prediction accuracy and produces interpretable models with variable selection and regularization^66^. The linear combination of selected features and the associated coefficients (obtained from LASSO) were considered as the radiomics score for prediction in that MRI modality.

For prediction models, we considered three categories of the prediction models. The models were constructed based on different combinations of clinical information and radiomics features (Fig. 2C). First, the baseline model was built with non-imaging characteristics including age, sex and ApoE genotype. A logistic regression classifier using the clinical variables was adopted. This model was built to establish a baseline model where only well-known clinical information was used to predict patient’s amyloid positivity. Second, we considered radiomics features from a single imaging modality. This approach assessed how different MRI modalities affected the prediction model. We also built a prediction model based on whole-brain cortical thickness as a variant of the single modality model. We replaced radiomics features with 148 regional cortical thickness values obtained from FreeSurfer and applied the same model building procedure. This model was built to compare our models with conventional approaches based on morphological features. Furthermore, our study constructed the combined models of (1) integrating the well-performing single modality models and (2) combining the first model with baseline clinical variables. All the features of the models were pooled together and the feature selection was applied again to remove the potential dependency between the combined models.

### Neuropsychological tests

For a comprehensive assessment of cognitive function, all subjects underwent the Korean version of the mini-mental state examination (K-MMSE) and the Seoul Neuropsychological Screening Battery, 2nd edition (SNSB-II). The SNSB-II evaluates many cognitive factors, including verbal and visual memory, visuoconstructive function, language, praxis, components of Gerstmann syndrome (acalculia, agraphia, right/left disorientation, finger agnosia), and frontal/executive functions. The detailed process of neuropsychological assessment was described in our previous work^67,68^.

### Statistics

In order to assess the classification performance of each model except the baseline model, a bootstrap approach was adopted splitting the original training data (n = 348) into training (70%) and testing (30%) sets 100 times while maintaining the ratio of amyloid positive and negative cases. In each iteration, the model building procedure was repeated from the training set and the constructed model was subsequently applied to testing and validation sets for performance evaluation. To assess the performance of the classifiers, mean and 95% confidence interval (CI) of AUC, sensitivity, and specificity were calculated. For evaluating the baseline model, the 95% CI of performance metrics were computed with results by 100 bootstrap procedures as well. We used Mann–Whitney U-test to compare AUC values and *p* values were adjusted for multiple comparisons using Bonferroni correction. AUC values of prediction models were compared with that of the baseline model, which uses age, sex, and ApoE genotype for amyloid positivity prediction, as well as with that of the T1 and T2 FLAIR radiomics model.

## Supplementary Information

Supplementary Information.
