# Supplementary material for: Predicting amyloid positivity in patients with mild cognitive impairment using a radiomics approach
Source: Sci Rep. 2021 Mar 26;11:6954. doi: 10.1038/s41598-021-86114-4 (PMC7997887; doi:10.1038/s41598-021-86114-4)
Supplement: Supplementary file 1 — Supplementary Information. [file 41598_2021_86114_MOESM1_ESM.docx]

**Predicting amyloid positivity in patients with mild cognitive impairment using a radiomics approach**

Jun Pyo Kim, MD^1,2,3†^, Jonghoon Kim, PhD^4†^, Hyemin Jang, MD^1,2,3^, Jaeho Kim, MD^1,2,3^, Sung Hoon Kang, MD^1,2,3^, Ji Sun Kim, MD^1,2,3^, Jongmin Lee, MD^1,2,3^, Duk L. Na, MD^1,2,3,7^, Hee Jin Kim, MD^1,2,3^, Sang Won Seo, MD^1,2,3,8,9*^, Hyunjin Park^5,6*^, PhD

† These authors contributed equally to this work

* These authors contributed equally to this work

^1^Department of Neurology, Samsung Medical Center, Seoul, Korea

^2^Samsung Alzheimer Research Center, Samsung Medical Center, Seoul, Korea

^3^Neuroscience Center, Samsung Medical Center, Seoul, Korea

^4^Department of Electronic and Computer Engineering, Sungkyunkwan University, Suwon, Korea

^5^Center for Neuroscience Imaging Research, Institute for Basic Science, Suwon, Korea

^6^School of Electronic and Electrical Engineering, Sungkyunkwan University, Suwon, Korea

^7^Department of Health Sciences and Technology, SAIHST, Sungkyunkwan University, Seoul, Korea

^8^Department of Clinical Research Design & Evaluation, SAIHST, Sungkyunkwan University, Seoul, Korea

^9^Center for Clinical Epidemiology, Samsung Medical Center, Seoul, Korea

**Address for correspondence:**

Sang Won Seo, MD PhD

Department of Neurology, Samsung Medical Center, Sungkyunkwan University, School of Medicine,

81 Irwon-ro, Gangnam-gu, Seoul, 06351, South Korea

Tel.: 82-2-3410-1397, Fax: 82-2-3410-0052

Email: [sangwonseo@empal.com](mailto:sangwonseo@empal.com)

Hyunjin Park, PhD

School of Electronic and Electrical Engineering, Sungkyunkwan University,

2066 Seobu-ro, Jangan-gu, Suwon-si, Gyeonggi-do, Republic of Korea

Tel: +82-31-299-4956, Fax: +82-31-290-5819

Email: hyunjinp@skku.edu

Supplement Table 1. Clinical characteristics of participants for the validation set (N=92)

|  | Overall (N=92) | Amyloid negative (N=48) | Amyloid positive (N=44) | p-value |
| --- | --- | --- | --- | --- |
| Age, mean (SD), years | 70.74 (9.28) | 71.56 (8.83) | 69.84 (9.76) | 0.377 |
| Male sex, No. (%) | 41 (44.6) | 19 (39.6) | 22 (50.0) | 0.427 |
| Education, mean (SD), years | 13.11 (4.21) | 12.42 (4.52) | 13.90 (3.71) | 0.094 |
| APOE e4 carrier, No. (%) | 36 (39.1) | 12 (25.0) | 24 (54.5) | 0.007 |
| MMSE score, mean (SD) | 26.21 (2.81) | 26.85 (2.44) | 25.50 (3.03) | 0.002^*^ |
| CDR-SOB, mean (SD) | 1.41 (0.83) | 1.20 (0.72) | 1.65 (0.89) | 0.002^*^ |
| Mean cortical thickness, mean (SD), mm | 2.39 (0.12) | 2.39 (0.14) | 2.39 (0.10) | 0.980^†^ |

Abbreviation: MMSE = Mini-Mental State Examination, CDR-SOB = Clinical Dementia Rating Scale Sum of Boxes

Data are presented as mean (standard deviation) for continuous variables and N (%) for categorical variables

* p-values were obtained from linear models, corrected for age, sex, and educational attainment

† p-values were obtained from linear models, corrected for age, sex, and intracranial volume

Supplement Table 2. Performances of the prediction models based on both standard radiomics and wavelet-based features.

|  | AUC | Sensitivity | Specificity |
| --- | --- | --- | --- |
| A. Test sets |  |  |  |
| *Single MRI modality* |  |  |  |
| T1 radiomics | 0.70(0.69 - 0.70) | 0.71(0.68 - 0.73) | 0.65(0.62 - 0.67) |
| T2 FLAIR radiomics | 0.70(0.69 - 0.71) | 0.81(0.79 - 0.83) | 0.57(0.55 - 0.59) |
| DTI radiomics | 0.67(0.66 - 0.68) | 0.69(0.67 - 0.72) | 0.63(0.61 - 0.65) |
| *Combined models* |  |  |  |
| T1 & T2 FLAIR radiomics | 0.71(0.71 - 0.72) | 0.73(0.70 - 0.75) | 0.65(0.62 - 0.67) |
| baseline + T1 & T2 FLAIR | 0.79(0.79 - 0.80) | 0.70(0.68 - 0.72) | 0.80(0.79 - 0.82) |
|  |  |  |  |
| B. Validation Set |  |  |  |
| *Single MRI modality* |  |  |  |
| T1 radiomics | 0.71(0.71 - 0.72) | 0.55(0.53 - 0.58) | 0.81(0.79 - 0.83) |
| T2 FLAIR radiomics | 0.66(0.64 - 0.67) | 0.68(0.65 - 0.72) | 0.65(0.63 - 0.68) |
| DTI radiomics | 0.63(0.63 - 0.64) | 0.60(0.56 - 0.63) | 0.67(0.65 - 0.70) |
| *Combined models* |  |  |  |
| T1 & T2 FLAIR radiomics | 0.69(0.69 - 0.70) | 0.66(0.63 - 0.69) | 0.69(0.66 - 0.71) |
| baseline + T1 & T2 FLAIR | 0.74(0.73 - 0.74) | 0.71(0.67 - 0.74) | 0.69(0.66 - 0.72) |

Abbreviations: AUC = Area under the curve, ApoE = Apolipoprotein E genotype, MRI = Magnetic resonance imaging, FLAIR = Fluid attenuation inversion recovery, DTI = diffusion tensor imaging

95% confidence intervals are presented in brackets


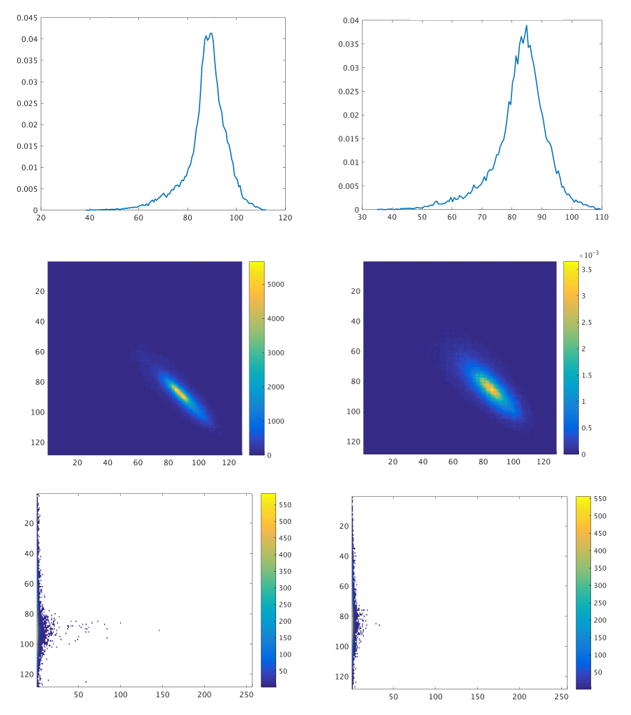


Figure S1. Representative histogram and data matrix for amyloid positive and negative cases using T1 images. The left subplots are the amyloid positive cases and the right subplots are the amyloid negative cases. The first row is the histogram, the second row is the gray-scale co-occurrence matrix, and the third row is the intensity size-zone matrix.


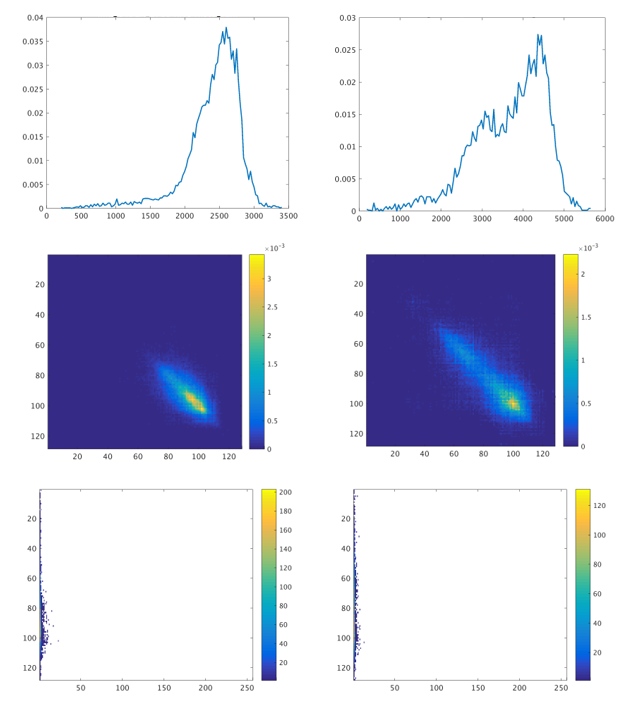


Figure S2. Representative histogram and data matrix for amyloid positive and negative cases using FLARI images. The left subplots are the amyloid positive cases and the right subplots are the amyloid negative cases. The first row is the histogram, the second row is the gray-scale co-occurrence matrix, and the third row is the intensity size-zone matrix.

Supplementary Table 3. Description of extracted radiomics features

| **Statistical based texture features** | | |
| --- | --- | --- |
| **First-order features : Histogram-based features** | | |
| **Parameter** | **Formula** | **Description** |
| Maximum | $Max=max(X\left( i \right))$  Where $X$ denote the 3d image matrix | Measures maximum intensity value of a histogram |
| Minimum | $Min=min(X\left( i \right))$  Where $X$ denote the 3d image matrix | Measures minimum intensity value of a histogram |
| Median | $Median=\frac{X(i)}{2}$  Where $X$ denote the 3d image matrix | Measures median intensity value of a histogram |
| Mean | $Mean=\frac{1}{N}\sum_{i}^{N} X(i)$  Where $X$ denote the 3d image matrix with $N$ voxel. | Measures mean intensity value of a histogram |
| Variance | $Variance=\frac{1}{N-1}\sum_{i=1}^{N} \left( X\left( i \right)-\bar{x} \right)^{2}$ | Measures squared distances of each value of a histogram from the mean |
| Energy | $Energy=\sum_{i}^{N} {X(i)}^{2}$  Where $X$ denote the 3d image matrix with $N$ voxel. | Measures squared magnitude value of a histogram |
| Standard deviation | $Std=\left( \frac{1}{N-1}\sum_{i=1}^{N} \left( X\left( i \right)-\bar{x} \right)^{2} \right)^{1/2}$  Where $X$ denote the 3d image matrix with $N$ voxel. | Measures amount of variation of a histogram. |
| Skewness | $Skewness=\frac{E{(x-\mu)}^{3}}{\sigma^{3}}$  Where $\mu$ is the mean of $x$, $\sigma$ is the standard deviation of $x$, $E$ is the expectation operator. | Measures asymmetry of a histogram. |
| Kurtosis | $Kurtosis=\frac{{E(x-\mu)}^{4}}{\sigma^{4}}$  Where $\mu$ is the mean of$x$, $\sigma$ is the standard deviation of $x$, $E$ is the expectation operator. | Measures “peakedeness” of a histogram (flatness of histogram) |
| Root mean square (RMS) | $RMS=\sqrt{\frac{1}{N}\sum_{n=1}^{N} \left\vert X_{n} \right\vert^{2}}$  Where $X$ denote the 3d image matrix with $N$ voxel. | Measures the square-root of the mean of the squares of the values of the histogram. This feature is another measure of the magnitude of a histogram |
| Inter quartile range | $IQR=Q_{3}-Q_{1}$  Where $Q_{3}$ denote the 3^rd^ quartile of histogram, $Q_{1}$ denote the 1^st^ quartile of histogram | Measures of variability, based on dividing a histogram into quartiles |
| Range | $Range=range(X\left( i \right))$ | Measures difference between the highest and lowest voxel values of a histogram |
| Entropy | $Entropy=-\sum_{i=1}^{N_{l}} P(i)\log_{2} P(i)$  Where $P$ denote the first order histogram with $N_{l}$ discrete intensity levels. | Measures irregularity of a histogram. |
| Uniformity | $Uniformity=\sum_{i=1}^{N_{l}} {P(i)}^{2}$  Where $P$ denote the first order histogram with $N_{l}$ discrete intensity levels. | Measures uniformity of a histogram. |
| Percentile | $Percentile=\left( \frac{n^{th} percentile}{100} \right) X(i)$ | Measures intensity value at the 2.5^th^ , 25^th^ ,50^th^ ,75^th^ , and 97.5^th^ percentile on histogram |
| **Higher order features : Gray-level Co-Occurrence Matrix (GLCM) based features** | | |
| Autocorrelation | $Autocorrelation =\sum_{i=1}^{N_{g}} \sum_{j=1}^{N_{g}} \mathrm{ij}\mathbf{P}(i,j)$ | Measures of the magnitude of the fineness and coarseness of texture |
| Cluster tendency | $Cluster tendency=$  $\sum_{i=1}^{N_{g}} \sum_{j=1}^{N_{g}} \left[ i+j-\mu_{x} -\mu_{y} \right]^{2}\mathbf{P}(i,j)$ | Measures of the homogeneity of GLCM |
| Maximum probability | $Maximum probability=max\{P\left( i,j \right)\}$ | Measures maximum value of GLCM matrix |
| Contrast | $Contrast=\sum_{i=1}^{N_{g}} \sum_{j=1}^{N_{g}} \left\vert i-j \right\vert^{2}\mathbf{P}(i,j)$ | Measures of the local intensity variation of GLCM |
| Difference entropy | $Difference entropy=\sum_{i=0}^{N_{g}-1} \mathbf{P}_{x-y}(i)\log_{2} [P_{x-y}(i)]$ | Measures entropy of processed GLCM matrix Px-y |
| Dissimilarity | $Dissimilarity=\sum_{i=1}^{N_{g}} \sum_{j=1}^{N_{g}} \left\vert i-j \right\vert\mathbf{P}(i,j)$ | Measures differences of entries in GLCM |
| Energy | $Energy=\sum_{i=1}^{N_{g}} \sum_{j=1}^{N_{g}} \left[ \mathbf{P}\left( i,j \right) \right]^{2}$ | Measures of the homogeneity of GLCM |
| Entropy | $Entropy=-\sum_{i=1}^{N_{g}} \sum_{j=1}^{N_{g}} \mathbf{P}(i,j)\log_{2} [\mathbf{P}\left( i,j \right)]$ | Measures irregularity of GLCM |
| Homogeneity1 | $Homogeneity1=\sum_{i=1}^{N_{g}} \sum_{j=1}^{N_{g}} \frac{\mathbf{P}\left( i,j \right)}{1+\left\vert i-j \right\vert}$ | Measures closeness of GLCM |
| Informational measure of correlation 1 (IMC1) | $IMC1=\frac{HXY-HXY1}{max\{HX,HY\}}$ | Secondary measure of Homogeneity1 |
| Variance | $Variance=\sum_{i=1}^{N_{g}} \sum_{j=1}^{N_{g}} \left( i-\mu_{x} \right)^{2}P(i,j)$ | Measures dispersion of the parameter values around the mean of the combinations of reference and neighborhood pixels |
| Sum average | $Sum average =\sum_{i=2}^{2N_{g}} \left[ iP_{x+y}\left( i \right) \right]$ | Measures the relationship between occurrences of pairs with lower and higher intensity values |
| Sum entropy | $Sum entropy =-\sum_{i=2}^{2N_{g}} P_{x+y}(i)\log_{2} \left[ P_{x+y}\left( i \right) \right]$ | Sum of neighborhood intensity value differences |
| Sum variance | $Sum variance=\sum_{i=2}^{2N_{g}} \left( i-SA \right)^{2}P_{x+y}(i)$ |  |
| Inverse variance | $inverse variance=\sum_{i=1}^{N_{g}} \sum_{j=1}^{N_{g}} \frac{P(i,j)}{\left\vert i-j \right\vert^{2}}, i\neq j$ |  |
| Inverse Difference Moment Normalized (IDMN) | $IDMN=\sum_{i=1}^{N_{g}} \sum_{j=1}^{N_{g}} \frac{P(i,j)}{1+\left( \frac{\left\vert i-j \right\vert^{2}}{N^{2}} \right)}$ | Measures the local homogeneity of an image |
| Where $\mathbf{P}\left( i,j \right)$is the gray level co-occurrence matrix for ($\delta=1, \alpha=0)$,  $N_{g}$is the number of discrete intensity value in the image,  $N$ is the number of voxels in the ROI,  $\mu$ is the mean of $\mathbf{P}\left( i,j \right),$  $p_{x}\left( i \right)=\sum_{j=1}^{N_{g}} \mathbf{P}(i,j)$ is the marginal row probabilities,  $p_{y}\left( i \right)=\sum_{i=1}^{N_{g}} \mathbf{P}(i,j)$ is the marginal column probabilities,  $\mu_{x}$ is the expected value of marginal row probability,  $\mu_{y}$ is the expected value of marginal column probability,  $\sigma_{x}$ is the standard deviation of $p_{x}$,  $\sigma_{y}$ is the standard deviation of $p_{y}$,  $p_{x+y}\left( k \right)=\sum_{i=1}^{N_{g}} \sum_{j=1}^{N_{g}} \mathbf{P}\left( i,j \right) , i+j=k, k=2,3,\ldots,2N_{g}$,  $p_{x-y}\left( k \right)=\sum_{i=1}^{N_{g}} \sum_{j=1}^{N_{g}} \mathbf{P}\left( i,j \right) ,\left\vert i-j \right\vert=k, k=0,1,\ldots,N_{g}-1$,  $HX=-\sum_{i=1}^{N_{g}} \mathbf{P}_{x}(i)\log_{2} \left[ p_{x}(i) \right]$ is the entropy of $\mathbf{P}_{x}$,  $HY=-\sum_{i=1}^{N_{g}} \mathbf{P}_{y}(i)\log_{2} \left[ p_{y}(i) \right]$ is the entropy of $\mathbf{P}_{y}$,  $HXY=-\sum_{i=1}^{N_{g}} \sum_{j=1}^{N_{g}} \mathbf{P}\left( i,j \right)\log_{2} \left[ \mathbf{P}(i,j) \right]$is the entropy of $\mathbf{P}\left( i,j \right)$  $HXY1=-\sum_{i=1}^{N_{g}} \sum_{j=1}^{N_{g}} \mathbf{P}\left( i,j \right)\log(p_{x}\left( i \right)p_{y}\left( j \right))$. | | |
| **Higher order features : Intensity size zone matrix (ISZM) based feature** | | |
| Size-zone variability | $\frac{1}{\Theta}{\sum_{m=1}^{M} \left[ \sum_{n=1}^{N} \mathbf{P}\left( m,n \right) \right]}^{2}$ | Variability in the size |
| Intensity variability | $\frac{1}{\Theta}{\sum_{n=1}^{N} \left[ \sum_{m=1}^{M} \mathbf{P}\left( m,n \right) \right]}^{2}$ | Variability in the intensity |
| Where $\boldsymbol{P}\left( m,n \right)$ is the intensity size zone matrix  $\Theta$ represents the number of homogeneous areas in tumor,  $M$ is the number of distinct intensity values,  $N$ is the size of homogeneous area in the matrix $\boldsymbol{P}\left( m,n \right)$ | | |

Supplementary Table 4. Frequently selected features

|  | Hippocampal features | Cortical features | non-imaging features |
| --- | --- | --- | --- |
| Single MRI modality |  |  |  |
| T1 | LHC-T1-Percentile Histogram 2.5  LHC-T1-Size Zone Variability  RHC-T1-Percentile Histogram 97.5 | LPC-T1-Std  LPC-T1-Percentile Histogram 97.5  LPC-T1-IMC1 | - |
| FLAIR | LHC-FLAIR-Std  LHC-FLAIR-Uniformity  LHC-FLAIR-Size Zone Variability  LHC-FLAIR-Autocorrelation  LHC-FLAIR-Contrast  RHC-FLAIR-GLCM-Energy  RHC-FLAIR-Std  RHC-FLAIR-IDMN  RHC-FLAIR-RMS  RHC-FLAIR-Autocorrelation | LPC-FLAIR-IDMN  LPC-FLAIR-IMC1  RPC-FLAIR-Std  RPC-FLAIR-Contrast  RPC-FLAIR-Intensity Size Zone Variability  RPC-FLAIR-Maximum Probability  RPC-FLAIR-Cluster Tendency  RPC-FLAIR-Sum Variance  RPC-FLAIR-Entropy | - |
| DTI | RHC-FA-Intensity Zone Variability | LPC-MD-Intensity Zone Variability | - |
| Combined models |  |  |  |
| T1 + T2 FLAIR | LHC-T1-Variance  LHC-T1-Autocorrelation  LHC-FLAIR-Uniformity  LHC-FLALRI-Autocorrelation  LHC-T1-Size Zone Variability  RHC-FLAIR-GLCM-Energy | LPC-T1-Maximum Probability  LPC-T1-Std  LPC-T1-Percentile Histogram 97.5  LPC-FLAIR-IDMN  LPC-T1-IMC1  RPC-FLAIR-IMC1 | - |
| Baseline+T1+FLAIR | LHC-T1-Percentile Histogram 2.5  LHC-FLAIR-Uniformity  LHC-FLAIR-Std  LHC-FLAIR-Autocorrelation  LHC-T1-Size Zone Variability  RHC-FLAIR-RMS | LPC-T1-IQR  LPC-T1-Intensity Zone Variability  LPC-T1-Sum Average  LPC-T1-Std  LPC-T1-Percentile Histogram 97.5  LPC-T1-IMC1  LPC-FLAIR-IDMN  RPC-FLAIR-IMC1 | ApoE |

Abbreviations: RHC, right hippocampus, LHC, left hippocampus, RPC, right precuneus, LPC, left precuneus, HIST, histogram, GLCM, gray-level co-occurrence matrix, IQR, interquartile range, FA, fractional anisotropy, MD, mean diffusivity, RMS, root mean square, IDMN, inverse difference moment normalized, IMC1, informational measure of correlation 1.
